# Methodology for High-Throughput Field Phenotyping of Canopy Temperature Using Airborne Thermography

**DOI:** 10.3389/fpls.2016.01808

**Published:** 2016-12-06

**Authors:** David M. Deery, Greg J. Rebetzke, Jose A. Jimenez-Berni, Richard A. James, Anthony G. Condon, William D. Bovill, Paul Hutchinson, Jamie Scarrow, Robert Davy, Robert T. Furbank

**Affiliations:** ^1^CSIRO Agriculture and FoodCanberra, ACT, Australia; ^2^High Resolution Plant Phenomics Centre, Australian Plant Phenomics Facility, CSIRO Agriculture and FoodCanberra, ACT, Australia; ^3^CSIRO Information Management and TechnologyCanberra, ACT, Australia; ^4^ARC Centre of Excellence for Translational Photosynthesis, Australian National UniversityCanberra, ACT, Australia

**Keywords:** field experiments, wheat, thermal imaging, image analysis, data processing, pixel histogram analysis

## Abstract

Lower canopy temperature (CT), resulting from increased stomatal conductance, has been associated with increased yield in wheat. Historically, CT has been measured with hand-held infrared thermometers. Using the hand-held CT method on large field trials is problematic, mostly because measurements are confounded by temporal weather changes during the time required to measure all plots. The hand-held CT method is laborious and yet the resulting heritability low, thereby reducing confidence in selection in large scale breeding endeavors. We have developed a reliable and scalable crop phenotyping method for assessing CT in large field experiments. The method involves airborne thermography from a manned helicopter using a radiometrically-calibrated thermal camera. Thermal image data is acquired from large experiments in the order of seconds, thereby enabling simultaneous measurement of CT on potentially 1000s of plots. Effects of temporal weather variation when phenotyping large experiments using hand-held infrared thermometers are therefore reduced. The method is designed for cost-effective and large-scale use by the non-technical user and includes custom-developed software for data processing to obtain CT data on a single-plot basis for analysis. Broad-sense heritability was routinely >0.50, and as high as 0.79, for airborne thermography CT measured near anthesis on a wheat experiment comprising 768 plots of size 2 × 6 m. Image analysis based on the frequency distribution of temperature pixels to remove the possible influence of background soil did not improve broad-sense heritability. Total image acquisition and processing time was *ca.* 25 min and required only one person (excluding the helicopter pilot). The results indicate the potential to phenotype CT on large populations in genetics studies or for selection within a plant breeding program.

## 1. Introduction

The gaseous exchange of water for carbon occurs at the stomata. From this exchange, plant surfaces, particularly leaves, are cooled by evaporation, and their temperatures typically decrease with increased evaporation. Stomatal closure and reduced transpiration manifest as a warmer canopy temperature (CT), while cooler CT is related to more open stomata and higher transpiration. Cooler CT has been associated with genetic gains in wheat yield, higher stomatal conductance, and maximum photosynthetic rates under non-water-limited conditions (Fischer et al., 1998). Similarly, cooler CT has been associated with increased grain yield in warm, irrigated conditions in Mexico (Reynolds et al., 1994; Amani et al., 1996; Ayeneh et al., 2002), and in a study comparing a selection of spring wheat cultivars from Australia and the International Maize and Wheat Improvement Center (CIMMYT) (Rattey et al., 2011). Similar findings were reported in water-limited environments, with cooler CT in wheat associated with increased yield (Blum et al., 1989; Rashid et al., 1999; Olivares-Villegas et al., 2007). When measured during grain-filling, cooler CT has been associated with increased rooting depth (Reynolds et al., 2007a), water use, and grain yield (Lopes and Reynolds, 2010). Conversely, warmer CT has been associated with conservative water use in different crops. In wheat, Pinter et al. (1990) reported that varieties with warmer CT in well-watered conditions had reduced stomatal conductance, used less water and were higher yielding when grown under water limitation.

Researchers have investigated the genetic basis underpinning CT in wheat using different populations. For example, Saint Pierre et al. (2010) studied five populations grown in three environments (water-limited, well-watered, and heat stress) and reported that gene effects were mostly additive with some dominance. Genetic mapping has revealed multiple quantitative trait loci for CT that are often pleiotropic with other important agronomic traits including yield and biomass (Pinto et al., 2010; Bennett et al., 2012; Mason et al., 2013; Rebetzke et al., 2013b). These studies generally report a strong association between cooler CT and yield, particularly when CT is measured during grain-filling. However, the polygenic control, together with the environmental sensitivity of stomatal conductance and CT (Rebetzke et al., 2013b), may reduce the heritability of the trait and hence the utility of CT for selection within a breeding program. Mason and Singh (2014) investigated CT as an indirect selection criterion for wheat under water limitation and heat stress environments. They concluded that the most useful application of CT within a breeding program would occur in the early generations, where yield testing is not performed and therefore indirect selection would be beneficial.

In the aforementioned studies, CT was measured with hand-held infrared thermometers. Use of hand-held instruments in large experiments is laborious, time-consuming and sensitive to weather fluctuations over short periods of time. Moreover, difficulties associated with maintaining a constant view angle and avoiding “contamination” from soil further complicate hand-held CT measurements. To address these issues, infrared thermography has been proposed as a method for CT phenotyping, owing to the advent of relatively affordable thermal cameras and user-friendly software for image processing (Jones et al., 2009; Takai et al., 2010; Prashar et al., 2013; Prashar and Jones, 2014). Recent studies have used unmanned aerial vehicles (UAVs) for the acquisition of thermal images for quantifying water stress in various field crops including cotton (Sullivan et al., 2007) and perennials including olives, mandarins, oranges, and apples (Berni et al., 2009a,b; Zarco-Tejada et al., 2012; Gómez-Candón et al., 2016). Chapman et al. (2014) demonstrated the use of UAV for various phenotyping applications including CT in sugarcane using thermal imaging.

For successful deployment of CT phenotyping within breeding programs, a scalable, and reliable methodology must first be developed and validated. Such a methodology must enable acquisition of CT from a large number of plots in a short time period (in the order of seconds), to reduce variance associated with weather fluctuations. The method must be accurate and precise to enable reliable and confident discrimination between genotypes. Moreover, the method must enable fast data acquisition and timely data processing. It must also be routine in delivery and readily accessible.

In this paper, we evaluate such a method developed for assessing CT on large field experiments. The method involves (i) airborne thermography from manned helicopter using a radiometrically-calibrated thermal camera to acquire CT data for large experiments in the order of seconds, and then (ii) data processing within minutes. The aim of this paper is to demonstrate the repeatability, scalability, and operative nature of the airborne thermography method for potential use in plot-scale phenotyping within a genetics study or within a plant breeding program.

## 2. Materials and methods

### 2.1. Field experiments

A field experiment containing contrasting wheat genotypes was grown in two successive years at the Managed Environment Facility (MEF) (Rebetzke et al., 2013a), located at Yanco (34.62°S, 146.43°E, elevation 164 m) in SE Australia. The soil at the Yanco MEF has been classified as chromosol and has a clay-loam texture (Isbell, 1996). The experiment was sown on 28th May in 2013 and 11th June in 2014 following canola or field pea break-crops and then managed with adequate nutrition and chemical controls as required for pest, weed, and leaf diseases. The experiments comprised 768 experimental plots, of size 2 × 6 m with 18 cm row spacing (orientated North-South), and included a range of germplasm that conformed to the following criteria described in Rebetzke et al. (2013a): contemporary high-yielding, elite germplasm with agronomically-acceptable flowering time and plant height, to minimize confounding variation in CT with canopy architecture.

Genotypes were sown into a partial-replicate design trial (average number of replicates was 1.4) at a sowing density of 200 seeds per m^2^. As described in Rebetzke et al. (2013a), two irrigation treatments (384 experimental plots per treatment) were used to simulate appropriate target environments, namely: *Treatment 1*, where irrigation was supplied to achieve a water limitation pattern close to the long-term climate median for the site and; *Treatment 2*, where irrigation was supplied to achieve the equivalent of a decile eight rainfall (wettest 20% of years) for the site. The mean grain yields for Treatment 1 and Treatment 2 were 2.2 and 2.0 t/ha in 2013 and 1.7 and 2.1 t/ha in 2014, respectively. For the majority of entries and for both treatments, the anthesis growth stage occurred between the 19th and 24th of September in 2013 and between the 24th of September and 2nd of October in 2014.

### 2.2. Hand-held thermography

Hand-held CT measurements were made by a single operator walking through the plots with an infrared thermometer (Mikron 1600, Mikron Infrared Instrument Co., Inc., Oakland, NJ, USA). To minimize capturing soil in the instrument's field-of-view, the infrared thermometer was held obliquely to each plot and scanned across the canopy at an angle of *ca.* 20° (above the horizontal) for *ca.* 4 s to derive an average CT-value for each plot (after Rebetzke et al., 2013b). Measurements were taken on the morning of 18th October 2013, between 11:00 and 11:30, (Treatment 2 only) and on the afternoon of 25th October 2013, between 13:50 and 14:20, (Treatment 2 only). The majority of entries in the experiment were in the grain-filling growth stage. Weather conditions on both days were sunny and clear, and winds light (≤20 km/h). Air temperature, relative humidity, and wind speed were recorded at a weather station located *ca.* 400 m from the experiment site. Weather conditions were only recorded prior to the commencement of hand-held CT measurements on the 18th October 2013, while on the 25th October 2013, weather conditions were recorded prior to the start of measurements and at the completion of measurements.

### 2.3. Airborne thermography

**Figure 1:**
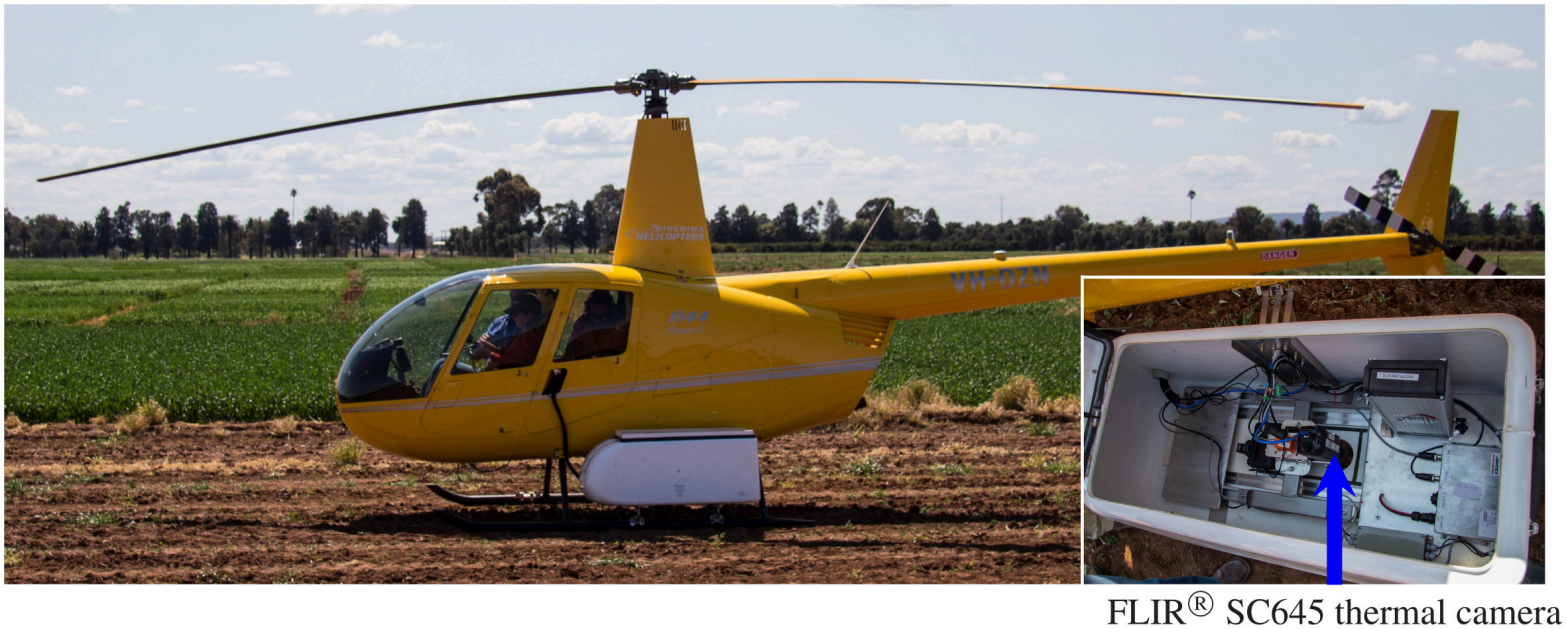
**Airborne thermography image acquisition system comprising a helicopter cargo pod with thermal camera and acquisition kit mounted on the skid of a Robinson R44 Raven helicopter**. Photo insert shows the inside of the helicopter cargo pod with arrow denoting FLIR® SC645 thermal camera: ±2°C or ±2% of reading; < 0.05°C pixel sensitivity; 640*x*480 pixels; 0.7 kg without lens.

**Figure 2:**
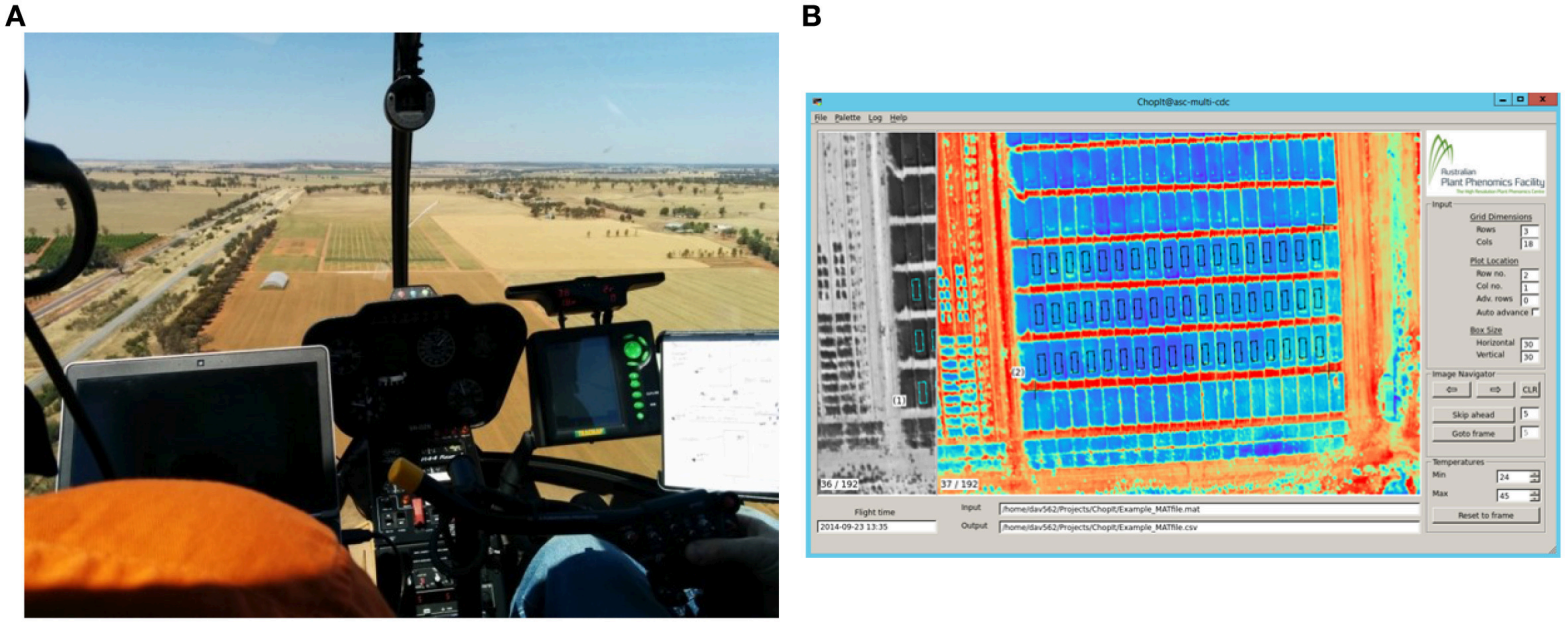
**Airborne thermography image acquisition and processing pipeline**. Total time to acquire and process images for an experiment comprising 1000 plots of size 2 × 6 m is *ca.* 25 min. **(A)** Image acquisition with helicopter. The images are recorded on a laptop and the passenger, left, provides real time assessment of the images and feedback to the pilot. This step takes < 10 s for an experiment comprising 1000 plots of size 2 × 6 m. **(B)** Screenshot of custom developed software called ChopIt. ChopIt is used for plot segmentation and extraction of CT from each individual plot for statistical analysis. This step takes *ca.* 20 min for an experiment comprising 1000 plots of size 2 × 6 m.

Airborne thermal images were acquired using the system described below on the 24th October 2013 at 10:00, 11:30, 12:30, and 13:30 and on the 2nd October 2014 at 09:00, 10:00, 11:00, 12:00, 13:00, and 14:00. On the 24th October 2013, the majority of entries in the experiment were in the grain-filling growth stage. On the 2nd October 2014, the majority of entries in the experiment were at anthesis. Weather conditions were sunny and clear, and winds light (≤20 km/h) on all days. The image acquisition and processing pipeline is depicted in Figures 1, 2, and the major steps are described below.

#### 2.3.1. Image acquisition

Thermal images were acquired using a thermal infrared camera (FLIR® SC645, FLIR Systems, Oregon, USA, for which the technical specifications are: ±2°C or ±2% of reading; < 0.05°C pixel sensitivity; 640 × 480 pixels; 0.7 kg without lens; 13.1 mm lens). The camera was mounted in a commercially-available helicopter cargo pod (R44 Helipod II Slim Line Top Loader, Simplex Aerospace, Oregon, USA) and fitted to a Robinson R44 Raven helicopter (Figure 1). Highly visible infra-red (IR) targets, made of black fabric and of size *ca.* 1 m^2^, were systematically positioned throughout the field to identify the experiment from adjacent collocated experiments. The IR targets were initially used for flight navigation and later for spatial referencing in post-processing of the thermal images. In contrast to other studies (e.g., Gómez-Candón et al., 2016), the IR targets were not used for temperature correction of the thermal images. Prior to acquiring thermal images, a GPS tracking line (an “AB line”) for subsequent flights was recorded by flying *ca.* 10 m above ground level (AGL) directly along the middle of the intended flight line.

In order to capture the experiment in a single flight pass whilst maximizing image resolution and avoiding motion blur, images were typically acquired at heights of 60 to 90 m AGL and at a flight velocity of 25–35 knots (45–65 km/h). Using the camera described above, at 60 m AGL, an image swath 43.6 by 32.1 m was obtained with a pixel size 7 × 7 cm, which equated to 204 temperature pixels per m^2^. At 90 m AGL, an image swath 65.3 by 48.1 m was obtained with a pixel size 10 × 10 cm, which equated to 100 temperature pixels per m^2^.

Thermal images were recorded on a laptop computer with FLIR® ResearcherIR™ software which was also used to control the camera. This proprietary software is provided for camera control and comprises basic image analysis features. The laptop and camera were manually operated by the helicopter passenger. Immediately prior to acquiring data for a particular experiment, the passenger would manually apply the shutter-based non-uniformity correction (NUC) and focus the camera, thereby ensuring image sharpness and that the NUC was not automatically applied during the run. Whilst acquiring thermal images, the passenger checked the images for complete coverage of the experiment using the IR targets and, in this way, provided real time assessment of the images and feedback on the helicopter flight path to the pilot (Figure 2A).

This method enabled capture of multiple high quality single images with at least 30% frame overlap in the direction of travel. Image acquisition with this system took < 10 s for the experiment described above comprising 768 plots.

#### 2.3.2. Image processing

The thermal images were pre-processed with FLIR® ResearcherIR™ software using the basic image analysis and processing features provided. Pre-processing included trimming of the image stack, to exclude extraneous images, and conversion from the RAW file format to Matlab (MAT) file format. This processing took *ca.* 2 min and was independent of experiment size.

Experimental plots were segmented from each thermal image using custom software developed with Python 2.7 (Python Software Foundation, https://www.python.org/); alias “ChopIt”. The ChopIt software works on a frame-by-frame basis extracting data from the raw imagery, whereby the user navigates through the image stack to ensure that each plot in the experiment has been sampled. A screenshot of the ChopIt graphical user interface is shown in Figure 2B. The ChopIt software is designed for semi-automated plot segmentation whereby the user controls the area sampled within plots by placement of bounding corners. The software also assigns a unique identifying number to each plot. The core geometric algorithm in ChopIt divides a four-sided region into a predefined number of rows and columns based on the placement of the bounding corners. The algorithm uses the concept of vanishing points and thus can accommodate situations where the image plane is not parallel to the ground. For a given row and column value, a plot rectangle is defined with a surrounding buffer, and the CT data are extracted from within the plot rectangle. The ChopIt software produces two output files comprising the CT data for each plot rectangle assigned by the user: (1) SQLite database file comprising all the CT pixel values for each experimental plot rectangle; and (2) an Excel file comprising a descriptive statistical summary for each experimental plot rectangle.

The process of plot segmentation and extraction of CT for each individual plot for statistical analysis took *ca.* 20 min for the experiment described above comprising 768 plots. Total image acquisition and processing time was *ca.* 25 min.

#### 2.3.3. Image quality control

**Figure 3:**
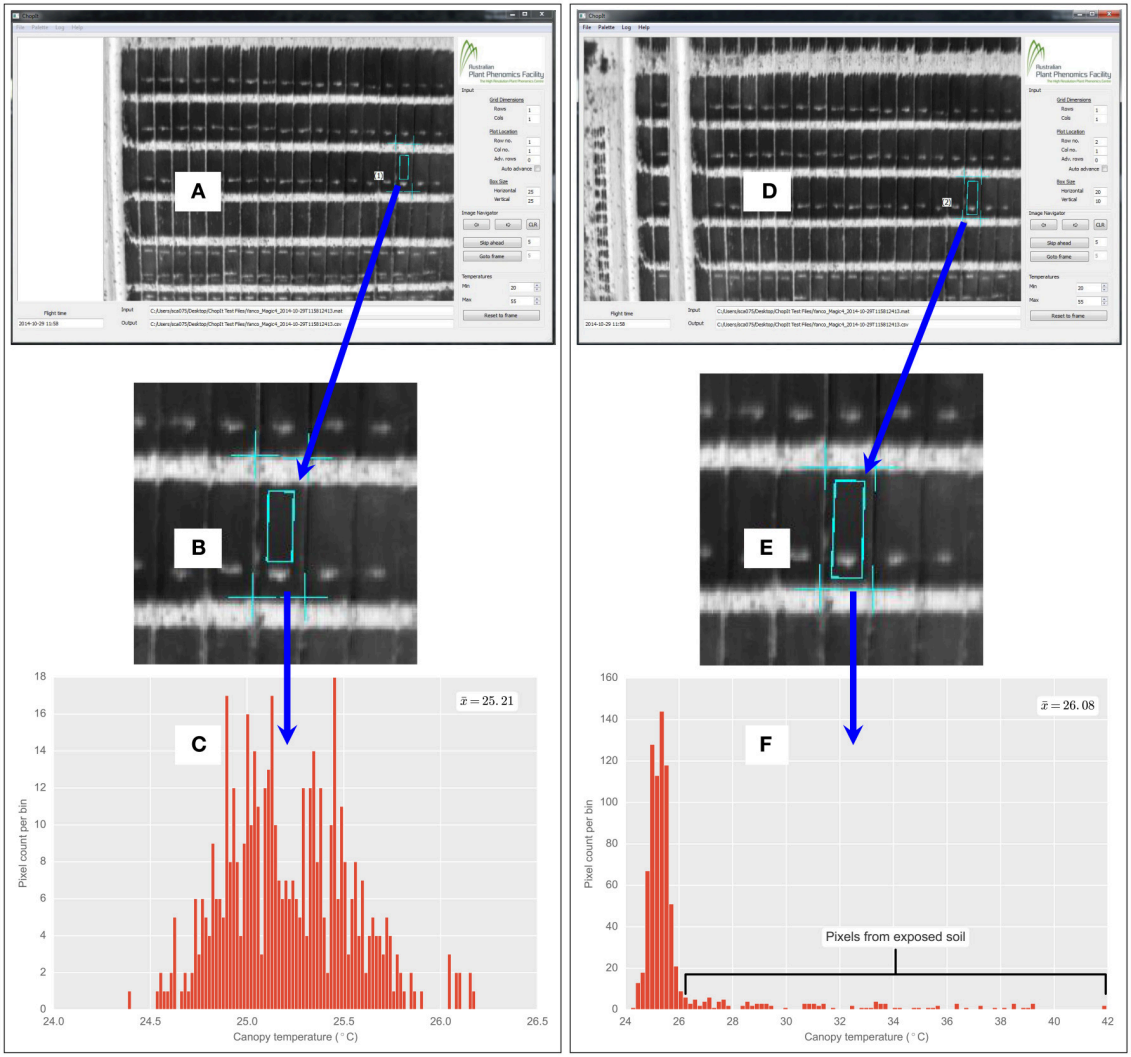
**Custom developed ChopIt airborne thermography image processing software**. Demonstration of quality control by user and flexibility of image analysis protocol, whereby the user can manually exclude exposed soil patches within a field plot. In this example, exposed soil patch is from biomass sample taken earlier on a 2 × 6 m field plot of wheat. **(A)** ChopIt user interface, where user has avoided exposed soil within the plot. **(B)** Magnified view showing exposed soil patch. **(C)** CT histogram is therefore void of pixels from the exposed soil patch. Compare with **(D)**, where for the same plot as **(A)**, user has included the exposed soil, evident in magnified view **(E)** and pixels from the soil patch are evident in the CT histogram **(F)**. Where $\bar{x}$ denotes the respective mean for **(C,F)**. The $\bar{x}$ from **(C)** is 0.87°C cooler than **(F)**.

The custom-developed ChopIt software provides a high level of quality control for the user to manually exclude poor quality sections of the plot or removed sections (e.g., where biomass cuts have been earlier sampled). This user-enabled flexibility in the image analysis protocol is demonstrated in Figure 3, where a section comprising a previous biomass sampling has been excluded on a plot with approximate dimensions of 2 × 6 m. In this fashion, sections of plots comprising biomass samples were excluded in the study reported herein. In addition to this feature of manually excluding poor quality sections of the plot during plot segmentation, post-processing of the temperature pixels is also possible, as all the pixel data for each plot are stored in a SQLite database file.

### 2.4. Analysis of the pixel frequency distribution

#### 2.4.1. Rationale

The water limitation imposed on the crop in the MEF can often result in incomplete crop ground cover. The incomplete ground cover may have implications for the airborne thermography measurements through the potential aggregation of crop canopy and the background soil temperatures, which in the case of dry soil is often warmer than the crop canopy. The potential for the background soil temperature to bias estimates of CT is exacerbated when the size of the image pixels is the same as, or greater than, the individual plant organs that comprise the crop canopy. In such cases, a pixel is likely to comprise both soil and plant canopy temperatures, thereby resulting in “mixed pixels”. The presence of mixed pixels is likely to bias the observed temperature toward the soil background temperature (Jones and Sirault, 2014).

In the airborne thermography system described above (Section 2.3), at an above-ground altitude of *ca.* 60 m, the pixel size is *ca.* 7 × 7 cm. This pixel resolution is several times greater than the leaf width of a typical wheat plant (*ca.* 1 cm) and, together with variation in plant establishment and canopy architecture, can result in mixed pixels and the need for image analysis to remove temperature pixels arising from the background soil that can bias the intrinsic measures of plant-based CT. Methods for handling thermal images containing mixed pixels were reviewed by Jones and Sirault (2014). These methods include automated thresholding algorithms such as the Otsu method (Otsu, 1979) and work best when discrete peaks are present in the histogram, representing multiple frequency distributions of temperature pixels.

**Figure 4:**
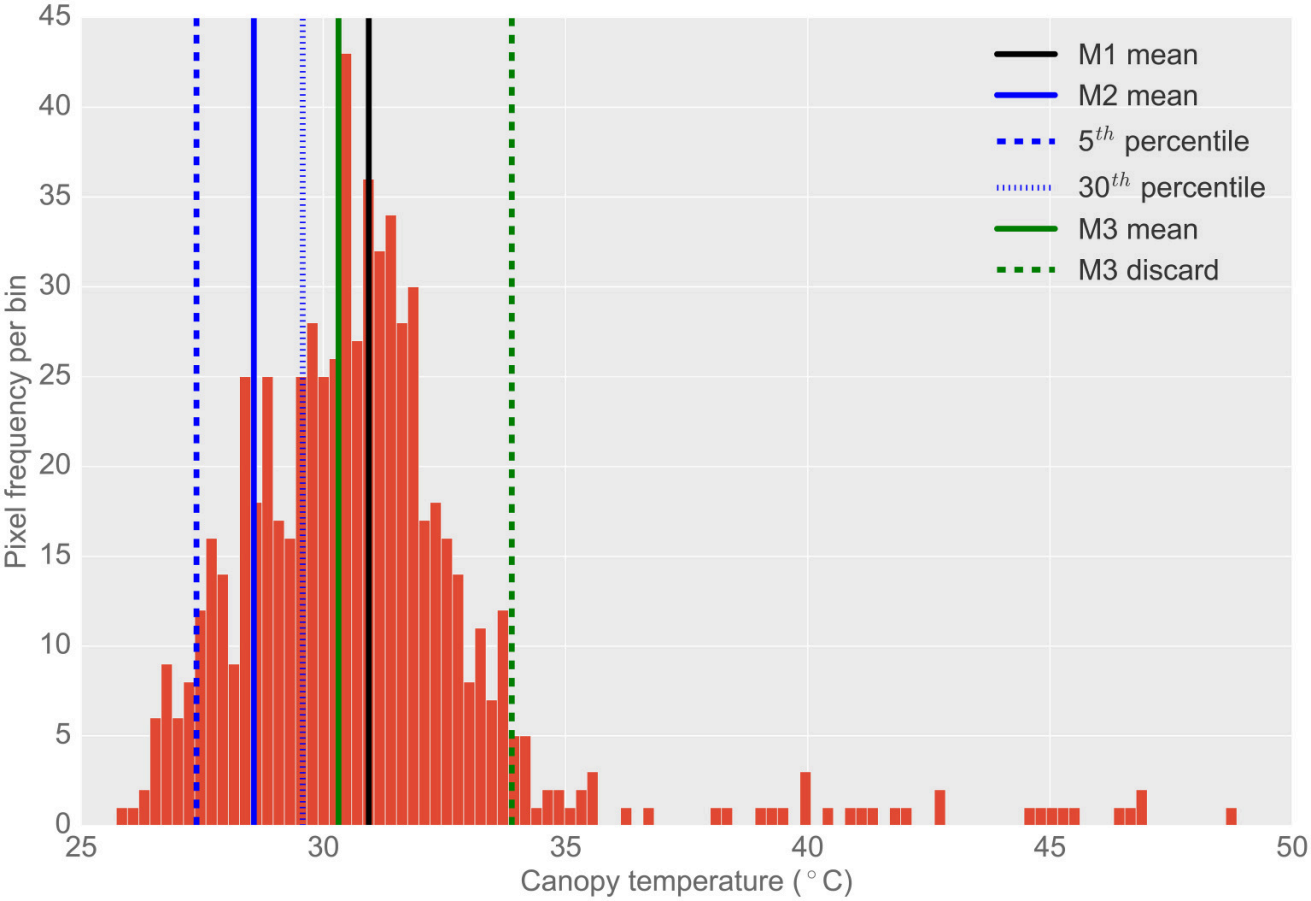
**Typical histogram of the frequency distribution of CT pixels from airborne thermography for a single 2 × 6 m experimental plot**. Different histogram trimming methods used to minimize bias from background soil temperature are shown. M1, The mean of all pixels (no pixels discarded). M2, The mean of coolest 25th percentile remaining after discarding coolest 5th percentile, designed to extract leaf temperature only. M3, Designed to discard warm pixels from soil patches resulting from poor establishment or biomass sampling. Total pixels in histogram (i.e., M1): 671. Total pixels using M2: 167. Total pixels using M3: 623. Difference between M1 and M2 was 2.4°C. Difference between M1 and M3 was 0.6°C. Difference between M3 and M2 was 1.7°C. Refer Section 2.4.2 for details on M1, M2, and M3.

A bimodal distribution with discrete peaks representing soil and plant canopy was not evident in the data acquired. Rather, pixel frequency distributions were unimodal with a long tail of warm temperature pixels, as shown in Figures 3, 4. The unimodal distribution may have resulted from the *ca.* 7 × 7 cm pixel resolution, whereby no clear difference between the mean temperature of the background soil and the mean temperature of the plant canopy was evident (Jones and Sirault, 2014). To account for the unimodal pixel distribution, the below-described methods of pixel frequency analysis were used.

#### 2.4.2. Methods for analysis of the pixel frequency distribution

The frequency distribution of the temperature pixels from a given plot rectangle produced from the ChopIt software was analyzed to determine if the observed temperature was biased by the background soil and whether this influenced the measurement repeatability. Three methods for analysis of pixel temperature bias were evaluated, depicted in Figure 4, and hereafter referred to as M1, M2, and M3:

1. M1: The mean of all pixels (no pixels discarded).
2. M2: The mean of the coolest 25th percentile remaining after discarding the coolest 5th percentile. This method was designed to extract the plant CT only. The percentile of pixels discarded and sampled were selected arbitrarily with the intention of maximizing the sampling of pixels arising from plant material and discarding mixed pixels and pixels comprising background soil. The coolest 5th percentile was discarded to avoid sampling background soil that might be cooler than the plant material (Jones, 2002; Jones and Sirault, 2014).
3. M3: A method designed to discard warm pixels arising from soil patches and calculated as follows:
  - For a given set of plot pixel temperatures, *x*, the mode of the distribution was estimated.
  - Then, a filter cut-off, *c*, was calculated according to: *c* = *min*(*x*) + 2(*mode*(*x*) − *min*(*x*))
  - The set, *x*, was then filtered by retaining only values where *x* < *c*.
  - The mean of this filtered set was then calculated.

From the representative example of pixel frequency distribution shown in Figure 4, the difference between M1 and M2 was 2.4°C, the difference between M1 and M3 was 0.6°C and the difference between M3 and M2 was 1.7°C.

### 2.5. Statistical analysis

CT data were analyzed after first checking for normality and error variance homogeneity at each date by time sampling event. Each event was analyzed separately with the best spatial models being determined after first fitting the experimental design and then modeling the residual variation with autoregressive row and column terms in the Genstat® statistical program (https://www.vsni.co.uk/software/genstat/). Significant spatial effects were identified and residuals assessed before determinations made to the need for fitting of other (e.g., linear) effects (Gilmour et al., 1997). Generalized heritabilities were then estimated after Holland et al. (2003).

## 3. Results

### 3.1. Hand-held thermography

**Figure 5:**
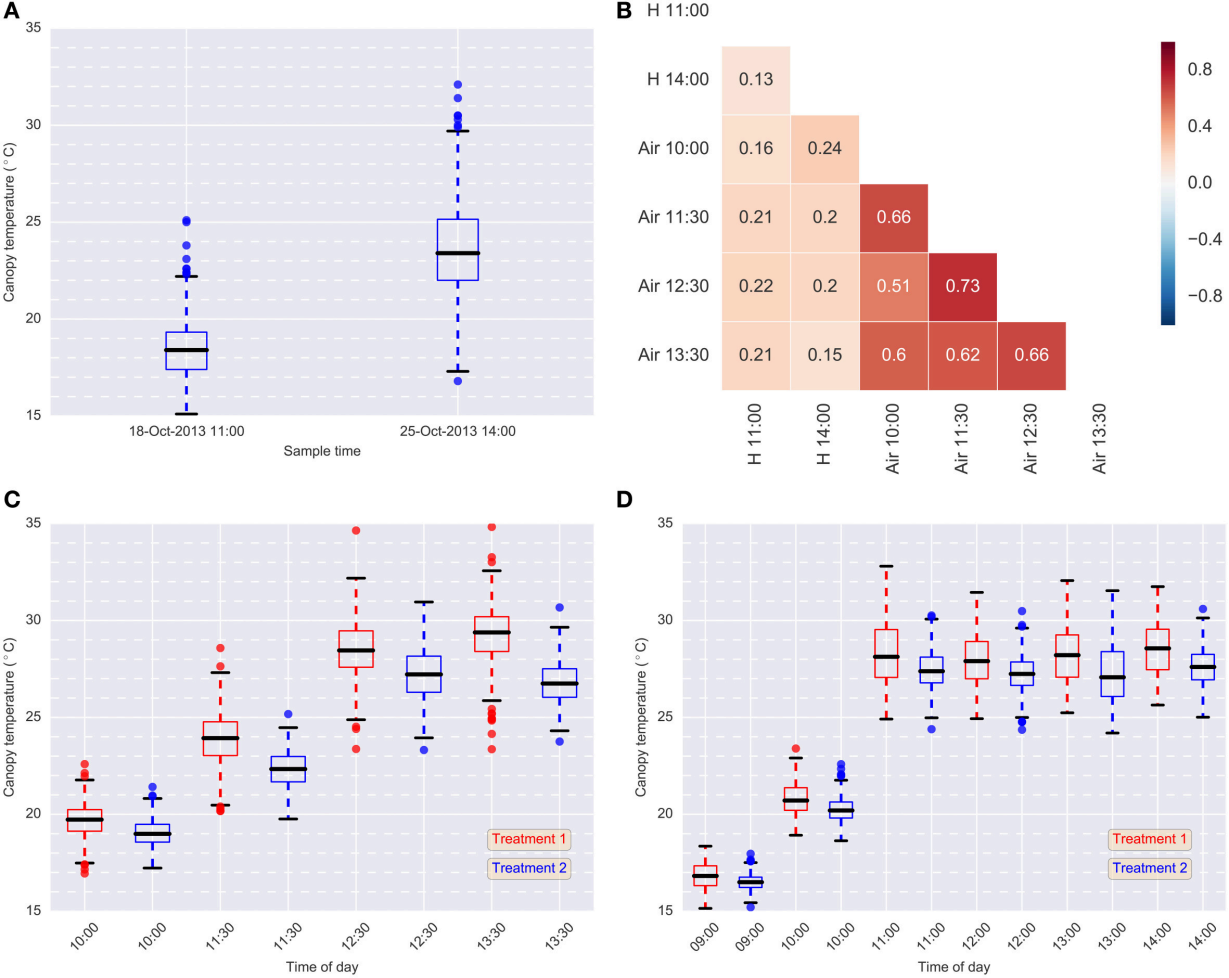
**Data summary**. **(A)** Box-plots of hand-held CT data for each sample event in 2013 (Treatment 2 only); **(B)** Pearson correlations between hand-held CT, H, (18-Oct-2013 11:00 and 25-Oct-2013 14:00) and airborne CT, Air, (24-Oct-2013) (Treatment 2); **(C)** box-plots of airborne thermography CT data for each flight time and treatment on 24-Oct-2013; **(D)** box-plots of airborne thermography CT data for each flight time and treatment on 2-Oct-2014. Each box-plot represents CT data from 384 experimental plots (384 experimental plots per treatment and 768 experimental plots in total). Airborne CT for each experimental plot was derived using M1: mean of all pixels (no pixels discarded). In **(C,D)**, Treatment 2 was consistently cooler than Treatment 1, owing to the greater water limitation applied to Treatment 1. In Treatment 2, irrigation was supplied to achieve the equivalent of a decile eight rainfall (wettest 20% of years) for the site, while in Treatment 1, irrigation was supplied to achieve a water limitation close to the long-term climate median for the site.

The results from the hand-held thermography are summarized in Table 1, and box-plots for each sample time are shown in Figure 5A. The range in plot CT was large in each sampling event. Broad-sense heritabilities for CT using the hand-held thermography method were 0.17 and 0.13 for the morning (18 October 2013) and afternoon measurements (25 October 2013), respectively (Treatment 2 only). The time taken to measure CT using hand-held thermography on 384 plots was *ca.* 30 min on both days. On the 25th October 2013, the air temperature changed from 17.8°C prior to the start of measurements to 19.0°C at the completion of measurements. At the same time, the relative humidity remained constant at 28%, and the wind speed remained constant at 17 km/h. Weather conditions were only recorded prior to the commencement of measurements on the 18th October 2013: air temperature was 11.9°C, relative humidity was 48% and wind speed was 11 km/h.

**Table 1:** **Summary of hand-held CT sampling events, weather conditions and resulting broad-sense heritabilities**.

**Date and time**	**Broad-sense heritability**	**Air temp. (°C)**	**Rel. humidity (%)**	**Wind speed (km/h)**
18 October 2013	0.17	11.9 (09:00)	48.0 (09:00)	11.0 (09:00)
11:00 to 11:30				
25 October 2013	0.13	17.8 (13:30)	28.0 (13:30)	17.0 (13:30)
13:50 to 14:20		19.0 (14:00)	28.0 (14:00)	17.0 (14:00)

Hand-held CT measurements were made on two occasions in 2013 on Treatment 2 only. In Treatment 2, irrigation was supplied to achieve the equivalent of a decile eight rainfall (wettest 20% of years) for the site. Treatment 2 comprised 348 plots. Values in parenthesis denote the time of day when air temperature, relative humidity, and wind speed were recorded. Note, that weather conditions were only recorded prior to the commencement of measurements on the 18th October 2013.

### 3.2. Airborne thermography

Box-plots summarizing the airborne thermography CT data for each flight time and irrigation treatment are shown for 2013 and 2014 in Figures 5C,D, respectively. Each box-plot represents CT data from 384 experimental plots (384 experimental plots per treatment and 768 experimental plots in total). The CT for box-plots shown in Figures 5C,D were derived from each experimental plot using M1, the mean of all pixels from a given plot rectangle produced from the ChopIt software with no pixels removed. For 2013 (Figure 5C) and 2014 (Figure 5D), Treatment 2 is consistently cooler than Treatment 1, owing to the greater water limitation in Treatment 1. Pearson correlations between the hand-held CT, obtained on the 18th October 2013 11:00 and the 25th October 2013 14:00, and the airborne thermography CT, obtained on the 24th October 2013, are shown in Figure 5B. The correlations between hand-held CT and airborne CT were <0.25.

**Figure 6:**
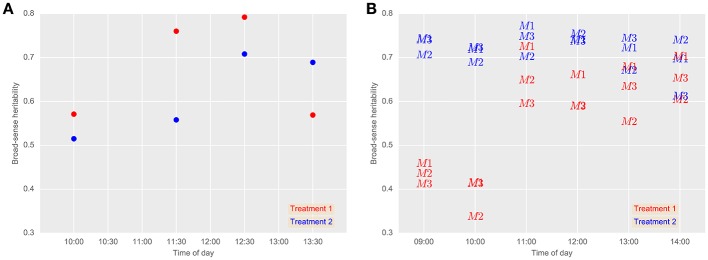
**Broad-sense heritabilities for airborne thermography CT for each flight event and treatment in 2013 (A) and 2014 (B). (A)** CT for each experimental plot was derived using M1: mean of all pixels (no pixels discarded). 24th October 2013 (grain-filling). **(B)** Comparison between airborne thermography pixel handling methods. 2nd October 2014 (anthesis). M1, mean of all pixels (no pixels discarded). M2, The mean of coolest 25th percentile remaining after discarding coolest 5th percentile, in order to extract plant CT only. M3, Designed to discard warm pixels from soil patches resulting from poor establishment or biomass sampling. Refer Section 2.4.2 for details on M1, M2, and M3. In Treatment 2, irrigation was supplied to achieve the equivalent of a decile eight rainfall (wettest 20% of years) for the site, while in Treatment 1, irrigation was supplied to achieve a water limitation close to the long-term climate median for the site. Generally, broad-sense heritability was high regardless of the pixel handling method used, except for early morning measurements on Treatment 1 in 2014.

Broad-sense heritabilities for airborne thermography CT for each flight time and irrigation treatment are shown for 2013 and 2014 in Figure 5. For the 2013 data, broad-sense heritability was calculated using CT derived from each experimental plot using M1 (Figure 6A). For the 2014 data, to test the influence of soil temperature bias on measurement repeatability, broad-sense heritability was calculated for CT estimated from M1, M2, and M3 (Figure 6B). With the exception of two early morning measurements on Treatment 1 in 2014, broad-sense heritability was high and ranged from 0.34 to 0.79. Figure 6B shows that for a given flight time, there was very little difference in broad-sense heritability for the three pixel handling methods. This result is in accordance with Figure S1, which shows the Pearson correlation calculated for all methods at each flight time. At any given flight time, all three pixel handling methods were highly correlated with Pearson correlations exceeding 0.86 and averaging 0.93. The background soil temperature did influence the observed CT but in this example, did not influence measurement repeatability (i.e., broad-sense heritability).

### 3.3. Analysis of the pixel frequency distribution

The aerial CT data incorporates influences from the background soil and the plant canopy. To investigate the significance of the effect of background soil, pairwise difference plots between M1, M2, and M3 (i.e., M1 and M2, M1 and M3, M3 and M2) were generated for airborne thermography data captured from Yanco MEF, 2nd October 2014, using the method of Bland and Altman (1986):

- Where *i* is a given set of temperature pixels derived at a particular time from a given plot rectangle produced from the ChopIt software described in Section 2.3.2.
- For a given pair, the mean of the pair as the abscissa (*x*-axis) value e.g., $\frac{M1_{i}\;+\;M2_{i}}{2}$.
- For a given pair, the difference between the two methods as the ordinate (*y*-axis) value e.g., *M*1_*i*_ − *M*2_*i*_.

The difference against mean plots are shown in Figure S2. For M1 and M2 (Figure S2A), and M3 and M2 (Figure S2C), the differences increased with time of day until 11:00 h, then from 12:00 to 14:00 h the differences decreased (M1 and M2 mean decreased 0.19°C). From the mean difference calculated across all sample times, M1 and M3 were on average 1.13°C and 0.94°C warmer, respectively, than M2. Further, M1 and M3 were as much as *ca.* 3.0°C warmer than M2 at sample times close to solar noon (11:00, 12:00, and 13:00 h). The majority of differences between M1 and M3 (Figure S2B) were close to zero and the mean difference across all sample times was 0.19°C.

## 4. Discussion

### 4.1. High broad-sense heritability obtained from airborne thermography methodology

The main finding reported herein is the large broad-sense heritability obtained for CT from the airborne thermography method, which contrasts with the low heritabilities reported with hand-held thermography sampling methods. Further, this was demonstrated in a large experiment comprising diverse wheat germplasm typical of a commercial wheat breeding program. Across both years, the broad-sense heritability for the airborne thermography ranged from 0.34 to 0.79, while for the hand-held infra-red thermometer, broad-sense heritability ranged from 0.13 to 0.17. Further, aside from two early morning measurements (09:00 and 10:00 h) on Treatment 1 in 2014, which ranged from 0.34 to 0.46, broad-sense heritability for the airborne thermography ranged from 0.52 to 0.79. The larger broad-sense heritabilities obtained from the airborne thermography can be attributed to the acquisition of thermal images of the entire experiment at effectively a single point in time, thereby overcoming confounding changes in local weather conditions during sampling to provide reliable assessment of CT for large experiments comprising hundreds of 10 m^2^ sized plots. Moreover, by measuring CT at effectively a single point in time, statistical analysis need only account for the spatial variation in CT, likely due to the below ground effects of soil structure and water availability, which can be accommodated by the experiment design and spatial analysis (Gilmour et al., 1997). In contrast, for the hand-held thermography method, the spatial analysis is confounded by temporal variation in weather conditions, which are more difficult to account for in the statistical analysis.

Broad-sense heritabilities obtained from the hand-held infra-red thermometer were small, ranging from 0.13 to 0.17, and typical of our experience with experiments of similar size previously undertaken at the Yanco site (data not shown). Further, Pearson correlations between the hand-held (18-Oct-2013 11:00 and 25-Oct-2013 14:00) and airborne thermography (24-Oct-2013) measures were <0.25, and the correlation between the two hand-held measurement events was low (0.13) (Figure 5B). It is likely that during the time required to measure all plots with the hand-held thermography method (*ca.* 30 min), the seemingly small changes in local weather conditions confounded the CT measurements, thereby resulting in low broad-sense heritabilities (Table 1). Another contributing factor might also be the range in area and canopy structure sampled by the user as they moved through the experiment. By contrast, an airborne thermography measurement of each plot in the entire experiment took approximately 3 s – a measurement of CT for 768 experimental plots at effectively a single point in time and at a common height above the ground. The heritability of hand-held CT can potentially be improved by using the time of sampling in the statistical analysis. For example, Rebetzke et al. (2013b) improved the heritability of hand-held CT by fitting “time of sampling” as a fixed linear effect in a mixed linear model.

To the best of our knowledge, heritability of CT is typically small on a single-plot basis and seldom reported in the literature. More commonly reported is heritability of CT estimated on a line-mean basis where multiple environments are included in the calculation. Heritabilities of CT calculated on a line-mean basis are often small to moderate in size for both diverse germplasm and related families such as recombinant inbred lines (RILs) and doubled-haploid (DH) lines. For example, Rebetzke et al. (2013b), using hand-held CT in three wheat populations containing 144–178 DH lines assessed in four irrigated environments, reported small narrow-sense heritabilities (0.12–0.32) on a single-plot basis and moderate to high line-mean heritability ranging from 0.38 to 0.91. Pinto et al. (2010) reported broad-sense heritability of 0.49 for CT measured during grain-filling on a RIL wheat population comprising 167 lines grown in six field experiments under drought and heat environments. In a separate study under similar environmental conditions, Lopes and Reynolds (2012) reported moderate broad-sense heritability on both a wheat population comprising 169 RILs (0.34) and 294 elite wheat lines from CIMMYT (0.38). Others have reported moderate line-mean heritability for diverse wheat germplasm calculated from studies comprising RILs in multiple environments (e.g., Reynolds et al., 2007b; Rattey et al., 2011; Lopes et al., 2012). In the above-mentioned studies, CT was measured using hand-held infrared thermometers. In the study reported herein, Figure 5 shows that the broad-sense heritability for the airborne thermography method, calculated on a single-plot basis, was typically >0.50 and as high as 0.79, which is considerably greater than literature reported calculations of CT heritability on both a single-plot and line-mean basis.

### 4.2. Analysis of the temperature pixel frequency distribution did not improve broad-sense heritability

In this study, methods based on filtering the frequency distribution of the temperature pixels to remove the influence of background soil did not improve broad-sense heritability (Figure 5). However, it is likely that the accuracy of the CT data was improved with the CT derived from M2. The difference against mean plots (Figure S2) show that for M1 and M2 (Figure S2A), and M3 and M2 (Figure S2C), the differences increased with the time of day until 11:00 h. This is possibly because the soil temperature increased more than the plant temperatures, thereby biasing the CT derived from M1 and M3. For M1 and M2, and M3 and M2, the decrease in differences from 12:00 to 14:00 h (for M1 and M2, the mean decreased 0.19°C) may have been due to the lower sun angle in the afternoon increasing the shaded portion of soil and thereby cooling it. That many of the differences between M1 and M3 (Figure S2B) were close to zero, indicates that M2 was more effective at deriving plant-based CT than M1 and M3. In contrast to M1 and M3, M2 was derived after discarding the warmest 70th percentile and it is therefore unlikely to be biased by the soil temperature, which, for dry soil, is likely to be warmer than the plant canopy. This approach of sampling cooler pixels, is likely to result in M2 more accurately approximating the actual plant CT than M1 and M3. The potential for improved CT accuracy may be beneficial in applications using energy balance equations to calculate stomatal conductance or transpiration, where soil-biased CT can lead to significant errors (Leinonen et al., 2006; Guilioni et al., 2008).

There may be phenotyping applications where the background soil could significantly reduce the accuracy and precision of CT measurements. For example: when multiple biomass samples have been taken from a plot leaving large areas of exposed soil; in plots with poor plant establishment; in early generation breeding trials or situations where seed number is limited and phenotyping is required on single plants or spaced rows; where row-spacing is too wide to completely cover the soil and in experiments that use raised beds with wide row spacings. To remove the influence of background soil, the custom developed ChopIt image processing software provides a high level of quality control to manually exclude poor quality sections of the plot or sections of the plot where biomass samples have been removed (Figure 3). In addition to this feature, post-processing based on the pixel frequency distribution (e.g., M2 and M3) is possible as all the pixels for a particular plot rectangle are stored in a SQLite database file.

### 4.3. Frame by frame image processing

The ChopIt software enables processing of the images on a frame-by-frame basis and was custom built for the application of field phenotyping of CT. Our image processing method contrasts with the widely used mosaicking method, where a large number of single frame images containing many plots are used to create a mosaic from which plot level information is extracted (e.g., Berni et al., 2009b; Chapman et al., 2014; Gómez-Candón et al., 2016). Mosaics were attempted with thermal images obtained from our image acquisition system. However, the use of mosaics presents a number of issues, namely: the fact that mosaicking software tends to modify the pixel's value in favor of the visual result; the mosaicking process is computationally intensive; and mosaicking requires accurate measurements of the external orientation of the images via the integration of the camera with a GPS and inertial measurement unit (IMU). For our application, processing thermal images on a single frame basis confers a number of advantages over mosaicking including: a reduction in image processing time; higher CT accuracy from working with original temperature values from the raw images without the application of any pixel interpolation or blending; and no mosaicking “black box”, which introduces another layer of measurement uncertainty to the process.

Conversely, the requirement to process the images on a frame by frame basis introduced a trade-off between encompassing the entire experiment in a single helicopter pass, whilst maximizing the pixel resolution by flying no higher than necessary. However, the requirement to encompass an entire experiment in a single pass conferred many advantages including reduced helicopter flight time and cost, faster image processing, reduced image processing errors, and the influence of changing weather conditions on the observed CT were minimized.

### 4.4. Unmanned aerial vehicles

Unmanned aerial vehicles (UAVs) and tethered balloons have also been used for the acquisition of thermal images in field phenotyping applications (e.g., Sullivan et al., 2007; Berni et al., 2009a,b; Jones et al., 2009; Zarco-Tejada et al., 2012; Chapman et al., 2014; Gómez-Candón et al., 2016). The smaller form and, in some jurisdictions, non-requirement for a licensed operator may enable opportunistic sampling on small experiments, whereas the manned helicopter system used in this study might otherwise be considered too expensive to hire or may not be locally available. However, UAVs are often limited to a small camera payload (e.g., 1.5–1.1 kg in Chapman et al., 2014 and 3.0 kg in Gómez-Candón et al., 2016); have limited endurance (e.g., 30–60 min in Chapman et al., 2014); are highly susceptible to wind; are often required to be operated within line of sight and sometimes require a license to operate. Moreover, the image mosaicking process often reported in the literature with UAVs necessitates multiple passes of the experiment to achieve sufficient image overlap. For example, Chapman et al. (2014) used a transect width of 10 m, while Gómez-Candón et al. (2016) used track and cross-track overlaps of 80 and 60%, respectively. As discussed above, such requirement for multiple passes increases the required flight time for a given experiment and increases the likelihood that changes in local weather conditions will confound to compromise measurements of CT. However, the ChopIt frame-by-frame image processing software could potentially be used with images acquired from a UAV platform, provided the image acquisition considerations described in Section 2.3.1 are adhered to.

In contrast to many UAVs described in the literature, the thermal image acquisition system used in this study, comprising a manned helicopter fitted with a helicopter cargo pod (Figure 1), has a payload limit of 45 kg. The large payload limit permits the use of a radiometrically-calibrated thermal camera with high accuracy and pixel to pixel sensitivity that negates the need for ground infra-red calibration targets and temperature correction during post-processing (Gómez-Candón et al., 2016). Together, these simplify the image processing. Moreover, the large payload provides the option to add more cameras and sensors if required for additional tasks and enables carriage of a high-capacity battery sufficient for several hours operating time. Further, the use of manned helicopter enables acquisition of CT measurements from multiple large field trials in a short time, which would otherwise require a UAV to fly beyond visual line-of-site, which is not permitted in some jurisdictions.

### 4.5. Potential for deployment of CT within commercial breeding programs

In breeder's trials, plot size is often smaller than those sown in this study (e.g., Rebetzke et al., 2014). A single 10 s pass of the helicopter-mounted thermal camera can capture up to 5000 individual 4 m^2^ plots in a breeder's yield trial. This application is ideally suited to the airborne thermography method, which we have shown readily scales up to experiments comprising 1000 individual 10 m^2^ plots. For 1000 plots of 2 × 6 m, acquisition of CT on a per plot basis using the airborne thermography and data handling described here takes *ca.* 25 min and aside from the helicopter pilot, requires only one person. The method could be used within a breeding program to assess spatial uniformity of yield experiments and provide guidance to appropriate statistical spatial models. The demonstrated link between CT and grain yield (Reynolds et al., 1994; Amani et al., 1996; Fischer et al., 1998; Ayeneh et al., 2002; Rattey et al., 2011; Rebetzke et al., 2013b) should provide opportunity to select for CT in early generation screening. For example, selection of CT in early generations was demonstrated in studies reporting reasonable genetic correlation between small plot CT, leaf porosity and full plot yield (Condon et al., 2004, 2007). In addition, augmenting breeder's visual selection with early generation measurements of CT can potentially identify a greater number of equally high yielding lines compared to breeder's visual selection alone (van Ginkel et al., 2004). Importantly, economic analysis indicates that the incorporation of CT measurements within a wheat breeding program is likely to provide an economic benefit (Brennan et al., 2007).

To assist uptake by breeders, several improvements in the airborne thermography method described here are possible, including: remote automation of the image acquisition process; use of a smaller manned helicopter to reduce the operating cost (e.g., Robinson R22 Raven helicopter); and use of GPS geo-referencing to improve image processing. Differences in canopy architecture that may influence CT could be accounted for by making use of measurements of fractional ground cover, from digital camera (e.g., Li et al., 2010), and canopy height that can now be routinely measured by ground-based LiDAR (e.g., Deery et al., 2014) but possibly aerial LiDAR in the future. Together, these potential improvements could reduce the cost per plot of the airborne thermography method.

The high helicopter operating cost, AU$1000/h, may prohibit the use of the airborne thermography method within some breeding (and research) programs. However, the cost per plot of the airborne method, on 3000 plots of size 10 m^2^, equates to AU$0.39 (*ca.* US$0.30) (Table S1), which is within 30% of the hand-held cost per plot reported by Brennan et al. (2007) (US$0.19 in 2007, which equates to US$0.22 in 2016 after adjusting for inflation). Given the similar cost per plot of the two methods, together with the greater repeatability of the airborne CT method compared with the hand-held CT method, the airborne CT method could be a cost-effective CT phenotyping method for use within breeding (and research) programs.

## 5. Conclusion

CT, as a surrogate measure for stomatal conductance and potentially photosynthesis, has been associated with genotypic variation in grain yield in numerous studies and therefore mooted as a possible phenotypic selection tool for use in genetics studies or in breeder's trials. For this to be realized, an inexpensive, scalable, and reliable CT methodology is required. The airborne thermography methodology described herein is such a method. The method is highly repeatable, as evidenced by the high broad-sense heritabilities obtained. The method is scalable: for an experiment comprising 768 plots of size 2 × 6 m, it takes *ca.* 25 min to obtain a CT measurement for each individual plot for statistical analysis. Moreover, the method requires only one person (not including the helicopter pilot) and utilizes purpose built image processing software for use by a non-technical user.

## Author contributions

All authors contributed to the conception of the study. GR designed the field experiment and undertook statistical analysis. PH, JS, and JJ designed and integrated the helicopter cargo pod system components. JS, PH, and JJ developed the image acquisition protocol. PH, RD, JS, JJ, and DD designed and built the ChopIt image processing software. DD, GR, JJ, RJ, AC, WB, RF contributed to the conception of the article. DD made the figures and wrote the paper with input and advice from the co-authors.

## Funding

This research was funded by the Australian Government National Collaborative Research Infrastructure Strategy (Australian Plant Phenomics Facility) and the Grains Research and Development Corporation (GRDC).

### Conflict of interest statement

The authors declare that the research was conducted in the absence of any commercial or financial relationships that could be construed as a potential conflict of interest.
