# Supplementary material for: Methodology for High-Throughput Field Phenotyping of Canopy Temperature Using Airborne Thermography
Source: Front Plant Sci. 2016 Dec 6;7:1808. doi: 10.3389/fpls.2016.01808 (PMC5138222; doi:10.3389/fpls.2016.01808)
Supplement: Supplementary file 1 [file DataSheet1.PDF]

Supplementary Material:
Methodology for high-throughput field phenotyping of canopy temperature using airborne thermography

David M. Deery*, Greg J. Rebetzke, Jose A. Jimenez-Berni, Richard A. James, Anthony G. Condon, William D. Bovill, Paul Hutchinson, Jamie Scarrow, Robert Davy, Robert T. Furbank

*Correspondence:
Author Name: David M. Deery
david.deery@csiro.au

1 TABLES

Table S1. Costings for the airborne thermography method at Yanco Managed Environment Facility (MEF). The costings are for one day, comprising two canopy temperature (CT) measurements (e.g. 11:30 and 12:30 h) on 3,000 plots of size 2 x 6 m. Costs charged by the High Resolution Plant Phenomics Centre for public sector research projects have been used, together with our experience at the Yanco MEF, where it typically takes 15 minutes to acquire images from 3,000 plots of size 2 x 6 m. Image processing time at the Yanco MEF, for one person, is 20 min/1,000 plots of 2 x 6 m.

Description	Comment	Cost
Helicopter hire[a]	R44, AU$1,000/h[b]	AU$2,000
Image acquisition[c]	15 min, 3,000 plots of 2 x 6 m	
	AU$500/h, 2 events (30 min)	AU$250
Image processing	20 min/1,000 plots	
	1 person for 2 hours, AU$30/h	AU$60
Total cost		AU$2,310
Cost per plot	Two CT measurements	AU$0.77
Cost per plot	Per CT measurement	AU$0.39

[a] Including helicopter return transit of 90 min from airport to the experiment site and 30 min of image acquisition on site.
[b] Minimum daily charge of AU$2,000 applies, equivalent to two hours of helicopter flying time.
[c] Thermal camera and helicopter cargo pod leased from the High Resolution Plant Phenomics Centre.

2 FIGURES

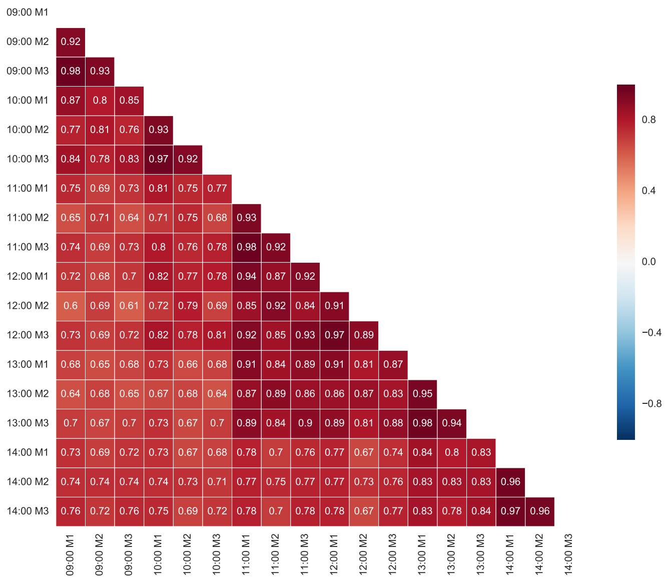

(1a) Treatment 1 ($n = 384$).

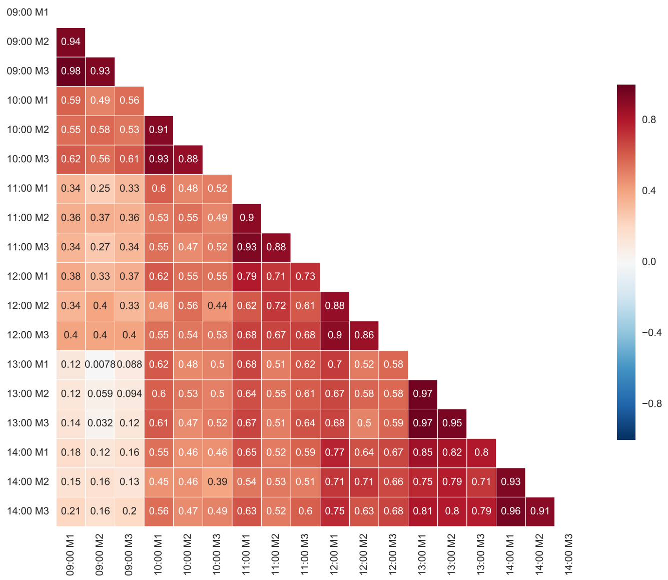

(1b) Treatment 2 ($n = 384$).

Figure S1. Pearson correlations for the three different airborne thermography pixel handling methods using data from 2nd October 2014 (treatment indicated). M1: The mean of all pixels (no pixels discarded). M2: The mean of coolest 25th percentile remaining after discarding coolest 5th percentile, in order to extract plant CT only. M3: Designed to discard warm pixels from soil patches resulting from poor establishment or biomass sampling. Refer section 2.4.2 for details on M1, M2 and M3. Note that for a given flight time, all three pixel handling methods were highly correlated and the Pearson correlation was ≥ 0.86 (mean 0.93).

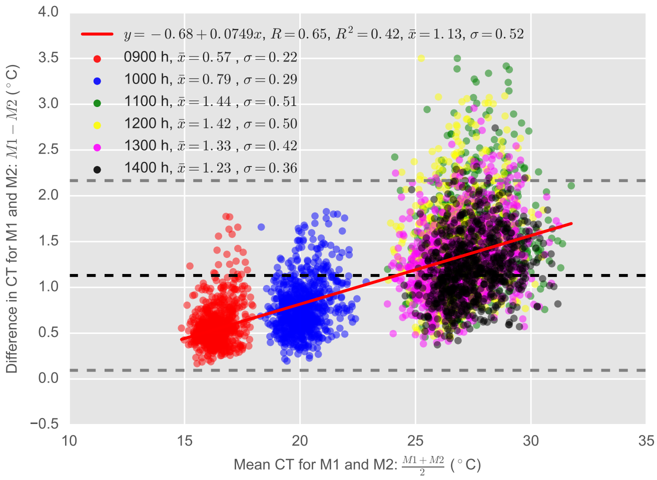

(2a) M1 and M2.

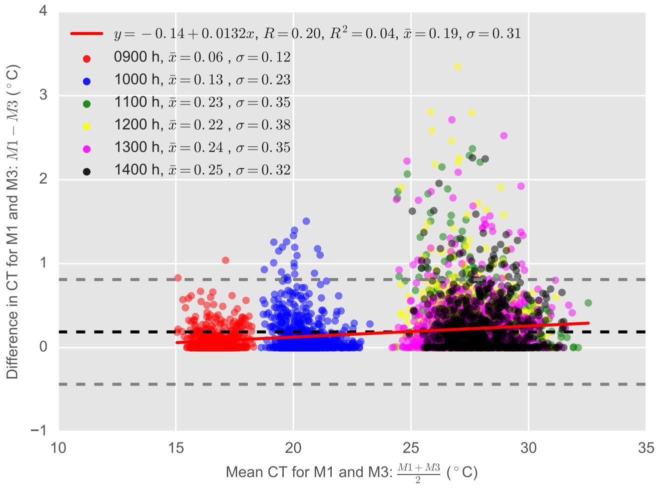

(2b) M1 and M3.

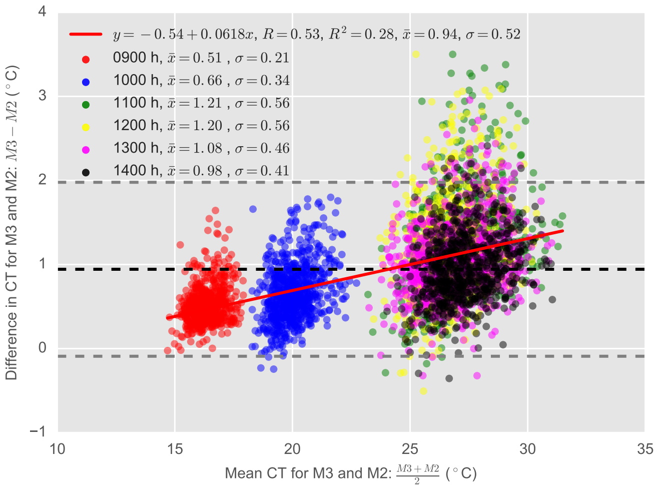

(2c) M3 and M2.

Figure S2. Airborne thermography difference against mean for the three pixel handling methods, described in section 2.4.2, using data from 2nd October 2014 (both treatments). M1 is the mean of all pixels (no pixels discarded). M2 is equal to the mean of coolest 25th percentile remaining after discarding coolest 5th percentile. M3 is designed to discard warm pixels from soil patches resulting from poor establishment or biomass sampling. Where $\bar{x}$ and σ denote the mean and standard deviation of the differences, respectively, for the respective flight time and across all flight times (adjacent to linear regression equation). Horizontal dashed lines are the mean (black) and plus / minus two standard deviations (grey) of the differences. For M1 and M2 (2a), and M3 and M2 (2c), the differences increase with time of day until 11:00 h, possibly because the soil temperature increased more than plant temperature and biased M1 and M3. For M1 and M2 (2a), and M3 and M2 (2c), the decrease in differences from 12:00 to 14:00 h (M1 and M2 (2a) $\bar{x}$ decreased 0.19°C) may have been due to the lower sun angle in the afternoon increasing the shaded portion of soil and thereby cooling it. While the soil temperature is unlikely to bias M2 and may result in a more accurate approximation of the actual plant CT. Many of the differences between M1 and M3 (2b) were close to zero.
